# Four patients with D-bifunctional protein (DBP) deficiency: Expanding the phenotypic spectrum of a highly variable disease

**DOI:** 10.1016/j.ymgmr.2020.100631

**Published:** 2020-08-15

**Authors:** Yuval E. Landau, Gali Heimer, Ortal Barel, Nechama Shalva, Dina Marek-Yagel, Alvit Veber, Elisheva Javasky, Aya Shilon, Andreea Nissenkorn, Bruria Ben-Zeev, Yair Anikster

**Affiliations:** a Metabolic Disease Unit, Edmond and Lily Safra Children's hospital, Sheba Medical Center, Sackler Faculty of Medicine, Tel Aviv University, Israel; b Metabolic Disease Unit, Schneider Children's Medical Center, Sackler Faculty of Medicine, Tel Aviv University, Israel; c Pediatric Neurology Unit, Edmond and Lily Safra Children's hospital, Sheba Medical Center, Sackler Faculty of Medicine, Tel Aviv University, Israel; d The Pinchas Borenstein Talpiot Medical Leadership Program, Chaim Sheba Medical Center, Tel Ha Shomer, Israel; e Genomic Unit, Sheba Cancer Research Center, Wohl Institute for Translational Medicine, Sheba Medical Center, Tel Hashomer, Israel; f The Child Development Center, Sheba Medical Center, Ramat Gan, Israel

**Keywords:** D-bifunctional protein, Peroxisomal, Neonatal seizures, Retinopathy

## Abstract

**Introduction:**

Peroxisomal D-bifunctional protein (DBP) deficiency is an autosomal recessive disorder historically described as a Zellweger-like syndrome comprising neonatal seizures, retinopathy, hearing loss, dysmorphic features, and other complications. The *HSD17B4* gene encodes DBP which is essential for oxidation of peroxisomal substrates. We describe 4 patients - 2 unrelated female girls and 2 monozygotic twin sisters - with DBP deficiency and phenotypic diversity.

**Patient reports:**

Patient 1 presented neonatally with hypotonia and seizures, and later on developed global developmental delay and regression, sensorineural hearing loss, nystagmus and cortical blindness. The brain MRI demonstrated bilateral peri-sylvian polymicrogyria. Whole exome sequencing revealed 2 mutations in the *HSD17B4* gene (c.752G>A, p.(Arg251Gln); c.868 + 1delG).

Patient 2 presented with hypotonia, motor delay, and sensorineural hearing loss in infancy, considerable developmental regression during her fourth year, nystagmus, and peripheral neuropathy. Brain MRI demonstrated cerebellar atrophy and abnormal basal ganglia and white matter signal, which appeared after the age of two years. Whole exome sequencing revealed 2 mutations in the *HSD17B4* gene (c.14 T>G, p.(Leu5Arg); c.752G>A, p.(Arg251Gln)).

Patients 3 and 4, two female monozygotic twins, presented with hypotonia, developmental delay, and macrocephaly from birth, and later on also sensorineural hearing loss, infantile spasms and hypsarrhythmia, and adrenal insufficiency. Brain MRI demonstrated delayed myelination, and an assay of peroxisomal beta oxidation suggested DBP deficiency. Sequencing of the *HSD17B4* gene revealed the same 2 mutations as in patient 1.

**Discussion:**

We describe 4 patients with variable and diverse clinical picture of DBP deficiency and particularly emphasize the clinical, biochemical, and neuroimaging characteristics. Interestingly, the clinical phenotype varied even between patients with the exact two mutations in the *HSD17B4* gene. In addition, in two of the three patients in whom levels of VLCFA including phytanic acid were measured, the levels were within normal limits. This is expanding further the clinical spectrum of this disorder, which should be considered in the differential diagnosis of every patient with hypotonia and developmental delay especially if accompanied by polymicrogyria, seizures, sensorineural hearing loss, or adrenal insufficiency regardless of their VLCFA profile.

## 1 Introduction

Peroxisomal D-bifunctional protein (DBP) deficiency (OMIM #261515) is an autosomal recessive disorder historically described as a Zellweger-like syndrome. It comprises of neonatal encephalopathy, seizure disorder, retinopathy, hearing loss, dysmorphic features accompanied by other systemic complications and in most cases infantile death [2,10]. The DBP is encoded by the *HSD17B4* gene and is essential for the oxidation of a wide range of peroxisomal substrates such as very long-chain acyl-CoAs and bile acid precursors [1,5,13].

The first DBP deficient patient was reported in 1989 [12], but only in 1999 was the defect located to the level of DBP [11]. The identification of additional patients followed thereafter, as well as the recognition that these patients resemble patients with severe peroxisomal biogenesis disorder. The prevalence of BPD deficiency is estimated to be 1:100,000, and as a result of its rarity, a solid understanding of its clinical and biochemical abnormalities, which is important for appropriate diagnosis and management, is lacking. Only a small number of patients has been described and molecularly confirmed [3].

In this work, we describe 4 patients from 3 families with variable phenotypes regarding their clinical, biochemical, and neuroimaging characteristics, which further expands the phenotypic spectrum from those that have been previously described.

## 2 Patient reports

### 2.1 Patient 1

The younger of two children to parents of mixed Jewish ethnicity with no consanguinity, was born at term after an uneventful pregnancy and delivery. She presented at her second day of life with severe hypotonia and refractory seizures, and consequently went through a comprehensive diagnostic process which revealed abnormal very-long-chain fatty-acid (VLCFA) profile suggestive of peroxisomal disorder (Table 1).

**Table 1:** Clinical, biochemical and molecular aspect of the patients. nt - not tested.

		Patient 1	Patient 2	Patient 3	Patient 4
Clinical	Age of onset	neonatal period	first months	neonatal period	neonatal period
	Age at last follow-up	3.9 years	9 years	8 months (death)	4 years
	Seizures	+	no	+ hypsarrhythmia	+ hypsarrhythmia
	Developmental delay	+	+	+	+
	Axial hypotonia	+	+	+	+
	Visual Abnormalities	+	no	+	+
	Hearing impairment	+	+	+	+
	Other complications	death	no	ARDS; death (8 months) Hepatic fibrosis	Adrenal insufficiency
	Brain MRI	1 week of age: suspected mild bilateral sylvian fissure cortical irregularities	At 5 years (but not at 2 years) cerebellar atrophy, diffuse abnormal signal in basal ganglia and in cerebral and cerebellar white matter	delayed white matter maturation, enlarged ventricles, and extra-axial fluid.	abnormal periventricular white matter signal, thin corpus callosum, mild cerebellar atrophy, and extra-axial fluid.
Biochemical	C22:0 [41.1–90.3 μM]	50.5; 39.2	60.1	nt	39
	C24:0 [37.4–74.9 μM]	67.9; 43	55.1	nt	−
	C26:0 [0.6–1.2 μM]	3.24; 1.18	1.07	nt	−
	C24:0/C22:0 [0.689–1.008]	1.345; 1.097	0.917	nt	0.93
	C26:0/C22:0 [0.011–0.022]	0.064; 0.03	0.018	nt	0.017
	Phytanic acid [0.3–11.5 μM]	0.1; 0.13	2	nt	3.7
	3-hydroxy-acylCoA dehydrogenase	nt	nt	nt	completely deficient
Molecular	Molecular analysis of the *HSD17B4* gene	c.752G>A p.(Arg251Gln) and c.868 + 1delG	c.14 T>G p.(Leu5Arg) and c.752G>A [p.Arg251Gln]	nt	c.752G>A p.(Arg251Gln) and c.868 + 1delG

**Fig. 1:**
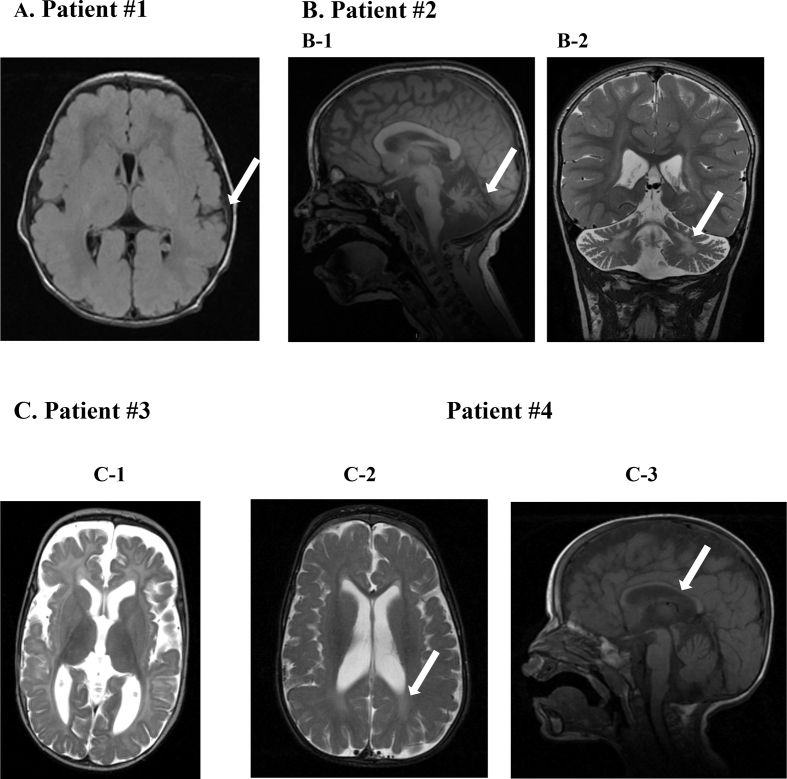
Brain MRI of the patients. A. An axial T2 Flair sequence of patient #1 at 6 days of age demonstrating mild bilateral peri-sylvian polymicrogyria (arrow). B. Patient #2 - MRI performed at 5 years. Significant cerebellar atrophy and mild cerebral atrophy are noted on the sagittal T1 view (B-1) and abnormal signal in the dentate and cerebral WM are demonstrated in the coronal T2 view (B-2) (arrows). C. Patients #3–4. C1) An axial T2 sequence of patient #3 at 8 months of age - delayed white matter maturation, enlarged ventricles, and extra-axial fluid; C-2) An axial T2 sequence of patient #4 at 18 months of age demonstrating abnormal periventricular white matter signal (arrow); C-3) A sagittal T1 sequence of patient #4 at 18 months of age showing thin corpus callosum (arrow), mild cerebellar atrophy, and extra-axial fluid.

Her brain magnetic resonance imaging (MRI) demonstrated mild bilateral peri-sylvian polymicrogyria (Fig. 1-A) and auditory brainstem evoked responses (ABR) test performed at 2 months of age was abnormal. Towards the end of the first year of life, she started exhibiting nystagmus and reduced visual responses. The seizures subsided around the age of 1 year but relapsed again at around 26 months. On her last follow up the age of 3 years and 9 months, she was suffering from multiple types of seizures (myoclonic, tonic and partial seizures), profound global developmental delay, sensorineural hearing loss, cortical blindness and nystagmus (Table 1) but normal fundi. Her neurological exam demonstrated severe axial and limb hypotonia, absent patellar deep tendon reflexes (DTR) and an extensor planter reflex. She succumbed at the age of 4 years and 1 month due to a viral respiratory illness. Targeted mutation analysis of the frequently Zellweger-associated PEX-1-related mutations was negative, however, whole exome sequencing (WES) revealed two presumably pathogenic mutations in the *HSD17B4* gene - c.752G>A, p.(Arg251Gln) and c.868 + 1delG. The c.752G>A mutation is present in the ClinVar database [8] where it is defined as uncertain significance. It was classified as pathogenic by 8 of the 9 prediction tools we used, and is extremely rare according to gnomAD databse (Table 2). The c.868 + 1delG mutation is present several times in the ClinVar database associated with DBP deficiency [3,7] and classified as pathogenic or likely pathogenic. This mutation causes an out of frame deletion of the splice site and its frequency in the gnomAD database is very low (Table 2). Both mutations segregated in the family with the disease. The mother, which is of a Jewish Iraqi / Tripolitan origin, was heterozygous to the c.752G>A mutation and the father, which is of a Jewish Moroccan / Halaby origin, was heterozygous to the c.868 + 1delG mutation.

**Table 2:** Description of the HSD17B4 mutations. NA - not available.

	Mutation 1	Mutation 2	Mutation 3
Genomic change (hg19, GRCh37)	g.chr5:118829525G > A	g.chr5:118829641delG	g.chr5:118788284 T > G
Transcript change – Varsome (NM_000414.4)	c.752G>A	c.868 + 1delG	c.14 T>G
Variant type	Missense	Splicing	Missense
Protein change	p.Arg251Gln	Intron 11 of 23 splicing	p.Leu5Arg
Allele frequency in gnomAD	0.00002393 (6/250,782 Alleles)	0.00001594 (4/250,944 Alleles)	0.00001196 (3/250,938 Alleles)
Prediction tool results			
SIFT	Damaging (0.003)	NA^a^	Damaging (0)
Polyphen2_HDIV	Damaging (0.991)	NA^a^	Damaging (0.946)
Polyphen2_HVAR	Pathogenic (0.735)	NA^a^	Damaging (0.823)
LRT	Deleterious (0.000001)	NA^a^	Deleterious (0.000001)
MutationTaster	Disease causing (0.9999)	NA^a^	Disease causing (0.9998)
MutationAssessor	Medium (3.445)	NA^a^	Low (0.84)
FATHMM	Damaging (−2.52)	NA^a^	Damaging (−2.62)
MetaSVM	Damaging (0.9151)	NA^a^	Damaging (0.6042)
MetaLR	Damaging (0.8405)	NA^a^	Damaging (0.7508)

a This mutation cannot be assessed by the above in silico prediction tools since it is a nonsense mutation. According to Varsome database it is defined as “pathogenic”.

### 2.2 Patient 2

An 11.5 years old girl, the eldest of two children to parents of mixed Jewish ethnicity with no consanguinity, was born at term after an uneventful pregnancy and delivery. She was noted to be hypotonic during the first few months of life and exhibited mild motor delay already from early development. At 2 years she was reported with hypotonia, moderate-severe sensorineural hearing loss and mild-moderate global developmental delay (could stand with support and had a few words). Workup included eye exam, MRI, EEG, echocardiogram and muscle biopsy, which were all within normal limits, as were basic genetic screening tests (karyotype, CGG repetitions in the FMR1 gene, and mutations screen for SMA and nemaline myopathy). At 4 years, when presenting to us for the first time, there was a history of gradual developmental regression over the past year (especially following febrile illnesses) with loss of gross motor and expressive language skills which were already acquired. Neurologic examination revealed nystagmus, severe axial hypotonia with paroxysmal hypertonicity, brisk reflexes, extensor planter response and clonus.

A comprehensive metabolic workup of blood, urine and CSF was uninformative containing also normal VLCFA profile including phytanic and pristanic acids. Electromyography demonstrated signs of axonal degeneration and a repeat MRI at the age of 5 years revealed cerebellar atrophy, mild cerebral atrophy and diffuse abnormal signal in the basal ganglia and cerebral and cerebellar white matter (Fig. 1-B). The neurodegenerative course accompanied with neuropathy and abnormal dentate signal on MRI was suggestive of infantile neuroaxonal dystrophy (INAD), however sequencing and deletion-duplication analysis of the PLAG26 gene were not revealing.

WES of the girl and her parents revealed two compound heterozygous mutations in the *HSD17B4* gene - c.752G>A, p.(Arg251Gln) which was found in patient 1, and c.14 T>G, p.(Leu5Arg). The later mutation was classified as pathogenic by 8 out of the 9 prediction tools we used, and is extremely rare according to gnomAD databse (Table 2). Both mutation segregated in the family with the disease. The mother, which is of a Jewish Syrian, Russian / Czechish, Polish origin, was heterozygous to the c.14 T>G mutation and the father, which is of a Jewish Tripolitan/Tunisian origin, was heterozygous to the c.752G>A mutation.

## 3 Patients 3–4

Two female monozygotic twins were born at 30 weeks in a normal delivery to unrelated parents of Jewish Moroccan origin. At birth they presented with severe hypotonia, macrocephaly and feeding difficulties, with delayed global development and sensorineural hearing loss diagnosed soon after at few months of age. At 3 months of age they started featuring partial seizures with secondary generalization and at 4 months they developed (only a day apart from each other) infantile spasms with hypsarrhythmia pattern on the electroencephalogram. Neurological examination demonstrated severe axial hypotonia with increased limb tone and reduced spontaneous movements. In addition, visual fixation and pursuit could not be elicited despite of normal funduscopic examination and visual evoked potentials (VEP) study.

An extensive blood, urine and CSF metabolic work-up was unrevealing. Specifically, tests for peroxisomal disorders, such as very long chain fatty acids including phytanic and pristanic acids and dihydroxy-acetone-phosphate acyltransferase (DHAPAT) activity in thrombocytes were normal. Also, lysosomal tests such as – arylsulfatase A, beta-galactosidase, hexosaminidase, galactocerebrosidase activity, and urine mucopolysaccharides (MPS) were within normal limits. Echocardiography was normal as well as an abdominal ultrasound. Electron microscopy did not show lipofuscin deposits in fibroblasts, and muscle biopsy showed normal histology and intact function of respiratory chain complexes.

Both twins were put on adrenocorticotropic hormone (ACTH) treatment for the infantile spasms at the age of 6 months with good response. Unfortunately, at 8 months one of the twins (patient 3) expired following a febrile illness that resulted in acute respiratory distress syndrome (ARDS) and multi-organ failure. Due to the paroxysmal increases in liver enzymes, a post mortem liver biopsy was carried out and was compatible with stage 3 fibrosis. In the remaining twin (patient 4), the ACTH was replaced with vigabatrin with relapse of the spasms and hypsarrhythmia within 3 months.

Brain MRI performed at 8 months in patient 3, and 12 and 18 months in patient 4 demonstrated extra-axial fluids, mild cerebral atrophy and delayed myelination while spectroscopy was normal (Fig. 1-C).

Repeat examination at 18 months in patient 4 demonstrated macrocephaly (H·C 51.5 cm, 3 SD above 50th percentile) with a large (2–2 cm) frontal fontanelle, bulky tongue, severe axial hypotonia with reduced spontaneous movements and a marked global developmental delay (does not roll over, does not reach for or hold objects, no babbling). Deep tendon reflexes could not be elicited, however, electromyography (EMG) and nerve conduction velocity (NCV) test were normal. Repeat Electroretinogram (ERG) and VEP were abnormal and consistent with retinal degeneration.

At the age of 4 years patient 4 developed adrenal insufficiency which had again raised the presumed diagnosis of a peroxisomal disorder. Consequently, a special test of the beta oxidation of peroxisomes in fibroblasts was performed (courtesy of Prof. Ronald J.A. Wanders) and demonstrated a normal activity of the hydratase component of the D-bifunctional enzyme whereas the activity of the 3-hydroxy-acylCoA dehydrogenase component was completely deficient. Finally, sequencing of the *HSD17B4* gene coding for the D-bifunctional protein, revealed that the girl harbors the same 2 compound heterozygous c.752G>A, p.(Arg251Gln) and c.868 + 1delG mutations found in patient 1, and parental segregation studies confirmed these 2 mutations to be in *trans*. The mother carries the c.868 + 1delG mutation and the father carries the c.752G>A mutation.

## 4 Discussion

**Fig. 2:**
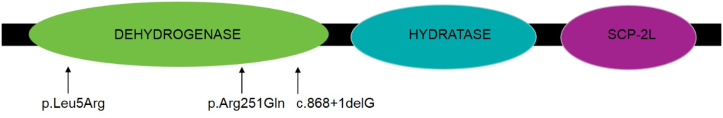
D-Bifunctional Protein structure and mutations. SCP-2 L. C-terminal sterol carrier protein 2-like domain.

Hydroxysteroid 17-Beta Dehydrogenase 4 (HSD17B4), also known as D-Bifunctional Protein (DBP), is a peroxisomal enzyme that catalyzes multiple steps of beta-oxidation of very long chain fatty acids. The DBP is comprised of 3 domains: the N-terminal short-chain alcohol dehydrogenase domain is encoded by exons 1–12 of the *HSD17B4* gene, the central 2-enoyl-CoA hydratase domain is encoded by exons 12–21, and the C-terminal sterol carrier protein 2-like domain (SCP-2 L) is encoded by exons 21–24 (Fig. 2) [3]. Recessive mutations in the *HSD17B4* gene are known to cause DBP-deficiency, an early-onset neurological disorder characterized by neonatal hypotonia, seizures, visual impairment, global developmental delay, and typical biochemical profile [2]. D-Bifunctional Protein deficiency are divided to 3 types: type I deficiency is generally associated with nonsense mutations, frameshift mutations, or in-frame deletions of 20 or more residues in the dehydrogenase domain. Type II deficiency is associated with missense mutations or in-frame deletions in the hydratase domain, and type III deficiency is associated with missense mutations or single amino acid deletions in the dehydrogenase domain [3].

The phenotypic of DBP deficiency spectrum is not completely clear yet. The common presentation is neonatal or infantile onset of a severe untreatable rapidly progressive multi-systemic disorder, although there were few reports of late onset and slowly progressive disease where the patients had normal metabolic profile and compound heterozygote mutations similar to our patients (e.g. hearing loss and neurodevelopmental symptoms and signs) [[4], [5], [6],^9^]. Two articles [2,3] were able to expand the phenotypic spectrum, both clinically, biochemically and molecularly and also establishing genotype-phenotype correlation to some extent. It has been shown that almost all children presented with neonatal hypotonia and seizures, died within the first 2 years of life without achieving any developmental milestones. However, few patients survived beyond the age of 2 years, five of which even had more prolonged survival (>7.5 years). In general, the less severely affected patients had predominantly more missense mutations compared with the more severely affected ones, who predominantly had mutations resulting in prematurely terminated protein due to structural anomalies affecting the DBP protein as a whole [3].

We describe here 4 patients from 3 families with DBP deficiency presenting variable phenotypes. While all the patients presented with hypotonia, sensorineural hearing loss, and global developmental delay or regression, they did not necessarily share other symptoms and signs (Table 1). Patient 1 demonstrated what commonly entitled as Zellweger syndrome spectrum presentation associated with neonatal onset of seizures, abnormal typical VLCFA profile and underlying congenital brain malformations. Patient 3–4 demonstrated more gradually developing infantile-onset disorder (rather than neonatal) with seizures first presenting on the third month of life and evolving into infantile spasms and hypsarrhythmia, and later on developed retinal degeneration, and adrenal insufficiency, while the VLCFA profile were normal as well as other parameters searching intentionally for a peroxisomal disorder including liver biopsy, and there was no brain malformation. Patient 2 presented slowly progressive and virtually more benign clinical picture first presenting with mild motor delay and hypotonia in the first months of life and further progressing to global developmental delay and sensorineural hearing loss during the next years. As she also had normal VLCFA profile, she initially was considered to have infantile neuroaxonal dystrophy (INAD) based on the neurodegenerative course accompanied with neuropathy and abnormal dentate signal on MRI, but this was molecularly ruled out before the final confirmation of DBP deficiency. To the best of our knowledge, the aforementioned combination of normal VLCFA levels in the context of significant multi-systemic involvement, further expanding the clinical-biochemical phenotypic spectrum in DBP deficiency.

The mutations described here are all located in the dehydrogenase domain, which could explain the normal hydratase activity found in patient 4 (Fig. 2). Mutations #1 and #3 are defined as missense and should thus cause type III deficiency and mutation #2 is defined as nonsense and should thus cause type 1 deficiency. The combination of two type III mutations in patient 2 can explain her relatively milder phenotype compared to patients 1,3 and 4 that are compound heterozygous to a type I mutation and a type III mutation. Thus, the genotypes do correlate to some extent with the clinical phenotype of the patients. Interestingly though, there are other aspects suggesting the genotypes do not completely correlate, such as the differences between patients 1 and the monozygotic twins (patients 3 and 4) who shared the two HSD17B4 mutations. In contrast to the very similar course of patients 3 and 4 (including appearance of hypsarrhythmia within a day apart of each other), patient 1 exhibited a categorically different phenotype in several aspects: age of onset, metabolic profile and presence of cortical malformation. This could suggest that other modifying factors might be involved in the underlying pathogenesis. The common Jewish Tripolitan origin of patient 1 mother and patient 2 father, both carrying the c.752G>A mutation might suggest an increased carrier rate of this mutation among Jewish Tripolitan population. Likewise, the common Jewish Moroccan origin of patient 1 father and patients 3 and 4 mother, both carrying the c.868 + 1delG mutation might imply an increased carrier rate of this mutation in Jewish Moroccan population. However, wide population screening is warranted in order to test this speculation. Although two of the mutations described here are present in the ClinVar database, to the best of our knowledge they have never been reported in the literature or HGMD database, thus further emphasizing the importance of reporting them here.

## 5 Summary

We describe here four patients from three families with DBP deficiency presenting variable clinical picture along the wide phenotypic spectrum of this disease from neonatal onset severe disease, through less severe infantile-onset disease, to more slowly progressive childhood-onset disease. In two of the three patients, in whom levels of VLCFA including phytanic acid were measured, the levels were within normal limits. Bearing in mind its variability, DBP deficiency should be considered in the differential diagnosis and work-up approach in any child, adolescent or adult with multi-systemic unexplained disorder, particularly characterized with hypotonia, hearing loss, neuro-developmental delay, seizures and visual impairment, even in the presence of normal VLCFA profile.
